# Supplementary material for: Computed Tomography-Based Radiomics for Long-Term Prognostication of High-Risk Localized Prostate Cancer Patients Received Whole Pelvic Radiotherapy
Source: J Pers Med. 2023 Nov 24;13(12):1643. doi: 10.3390/jpm13121643 (PMC10744672; doi:10.3390/jpm13121643)
Supplement: Supplementary file 1 [file jpm-13-01643-s001.zip › jpm-2726915-SI.pdf]

Supplementary Materials

Table S1. Definitions of clinical target volumes (CTVs) and planning target volumes (PTVs).

Organ	Target	Definition
**Prostate**	**$CTV_{prostate}$**	Included the entire prostate gland for all patients with the addition of proximal two-thirds of seminal vesicles for patients with intermediate- or high-risk prostate cancer.
	**$PTV_{prostate}$**	An anisotropic expansion of $CTV_{prostate}$ with a 6-mm margin posteriorly and 1-cm margin in all other directions.
**Lymph nodes**	**CTV_{LN}**	Included the distal common iliac lymphatics, external iliac lymphatics, internal iliac lymphatics, presacral lymphatics and obturator lymphatics.
	**PTV_{LN}**	An isotropic expansion of CTV_{LN} with a 5-mm margin.

Table S2. Acceptance criteria and organs at risk constraints.

Volume	Acceptance criteria or constraints
PTV	$Dose_{mininum}$ ≥ 95% of prescribed dose<br>≤ 3% received > 107% of prescribed dose<br>≤ 3% received < 93% of prescribed dose
Rectum	$Volume_{75Gy}$ ≤ 15%<br>$Volume_{70Gy}$ ≤ 20%<br>$Volume_{65Gy}$ ≤ 25%<br>$Volume_{60Gy}$ ≤ 35%<br>$Volume_{50Gy}$ ≤ 50%
Bladder	$Volume_{80Gy}$ ≤ 15%<br>$Volume_{75Gy}$ ≤ 25%<br>$Volume_{70Gy}$ ≤ 35%<br>$Volume_{65Gy}$ ≤ 50%
Femoral head	$Volume_{50Gy}$ ≤ 50%
Bowel space	$Volume_{45Gy}$ ≤ 200 cc
Penile bulb	$Dose_{70\%}$ ≤ 70 Gy<br>$Dose_{90\%}$ ≤ 50 Gy
Normal tissue	$Dose_{maximum}$ ≤110% of prescribed dose

Table S3. Number of extracted radiomic features.

	Extracted radiomic features (n=651)						
	Shape	First-order	Texture				
			GLCM	GLSZM	NGTDM	GLRLM	GLDM
Original	14	18	0	0	0	0	0
Discretized	0	0	22	16	5	16	14
$LoG_{0.5mm}$	0	18	0	0	0	0	0
LoG_{2mm}	0	18	0	0	0	0	0
LoG_{3mm}	0	18	0	0	0	0	0
LoG_{4mm}	0	18	0	0	0	0	0
$LoG_{4.5mm}$	0	18	0	0	0	0	0
LoG_{5mm}	0	18	0	0	0	0	0
$LoG_{0.5mm}$ + Discretized	0	0	22	16	5	16	14
LoG_{2mm} + Discretized	0	0	22	16	5	16	14
LoG_{3mm} + Discretized	0	0	22	16	5	16	14
LoG_{4mm} + Discretized	0	0	22	16	5	16	14
$LoG_{4.5mm}$ + Discretized	0	0	22	16	5	16	14
LoG_{5mm} + Discretized	0	0	22	16	5	16	14
**Sub-total**	**14**	**126**	**154**	**112**	**35**	**112**	**98**

Shape features (3-dimensional)	
1. Mesh volume	8. Maximum 2D diameter (column)
2. Voxel volume	9. Maximum 2D diameter (row)
3. Surface area	10. Major axis length
4. Surface area to volume ratio	11. Minor axis length
5. Sphericity	12. Least axis length
6. Maximum 3D diameter	13. Elongation
7. Maximum 2D diameter (slice)	14. Flatness

First-order features	
1. Energy	10. Interquartile range
2. Total energy	11. Range
3. Entropy	12. Mean absolute deviation
4. Minimum	13. Robust mean absolute deviation
5. 10th percentile	14. Root mean squared
6. 90th percentile	15. Skewness
7. Maximum	16. Kurtosis
8. Mean	17. Variance
9. Median	18. Uniformity

Gray level co-occurrence matrix (GLCM) features	
1. Auto correction	12. Joint entropy
2. Joint average	13. Informational measure of correlation 1
3. Cluster prominence	14. Informational measure of correlation 2
4. Cluster shade	15. Inverse difference moment
5. Cluster tendency	16. Inverse difference moment normalized
6. Contrast	17. Inverse difference
7. Correlation	18. Inverse difference normalized
8. Difference average	19. Inverse variance
9. Difference entropy	20. Maximum probability
10. Difference variance	21. Sum squares
11. Joint energy	22. Sum entropy

Gray level size zone matrix (GLSZM) features	
1. Small area emphasis	9. Zone variance
2. Large area emphasis	10. Zone entropy
3. Gray level non-uniformity	11. Low gray level zone emphasis
4. Gray level non-uniformity normalized	12. High gray level zone emphasis
5. Size-zone non-uniformity	13. Small area low gray level emphasis
6. Size-zone non-uniformity normalized	14. Small area high gray level emphasis
7. Zone percentage	15. Large area low gray level emphasis
8. Gray level variance	16. Large area high gray level emphasis

Neighbouring gray tone difference matrix (NGTDM) features
1. Coarseness
2. Contrast
3. Busyness
4. Complexity
5. Strength

Gray level run length matrix (GLRLM) features	
1. Short run emphasis	9. Run variance
2. Long run emphasis	10. Run entropy
3. Gray level non-uniformity	11. Low gray level run emphasis
4. Gray level non-uniformity normalized	12. High gray level run emphasis
5. Run length non-uniformity	13. Short run low gray level emphasis
6. Run length non-uniformity normalized	14. Short run high gray level emphasis
7. Run percentage	15. Long run low gray level emphasis
8. Gray level variance	16. Long run high gray level emphasis

Gray level dependence matrix (GLDM) features		
1. Small dependence emphasis	6. Gray level variance	11. Small dependence low gray level emphasis
2. Large dependence emphasis	7. Dependence variance	12. Small dependence high gray level emphasis
3. Gray level non-uniformity	8. Dependence entropy	13. Large dependence low gray level emphasis
4. Dependence non-uniformity	9. Low gray level emphasis	14. Large dependence high gray level emphasis
5. Dependence non-uniformity normalized	10. High gray level emphasis	

Figure S1. Shape features, first-order features and texture features extracted from the $CTV_{prostate}$. Texture features include grey level co-occurrence matrix (GLCM) features, grey level size zone matrix (GLSZM) features, neighbouring grey tone difference matrix (NGTDM) features, grey level run length matrix (GLRLM) features and grey level dependence matrix (GLDM) features.
